# Time-Restricted Eating Is Feasible and Acceptable and Improves Glycemic Control in Veterans with Spinal Cord Injury and Obesity: A Pilot Trial

**DOI:** 10.3390/nu18172839

**Published:** 2026-08-29

**Authors:** Geoffrey V. Henderson, Chenyu Liu, Liangliang Zhang, Malek El Muayed, Krista A. Varady, Courtney M. Peterson, David A. Levitsky, Daniel E. Forman, Steven W. Brose

**Affiliations:** 1 Syracuse Department, Veterans Affairs Medical Center, Syracuse, NY 13210, USA; 2 Department of Physical Medicine and Rehabilitation, SUNY Upstate Medical University, Syracuse, NY 13210, USA; 3 Cleveland FES Center, Cleveland, OH 44106, USA; 4 Department of Population and Quantitative Health Sciences, School of Medicine, Case Western Reserve University, Cleveland, OH 44106, USA; 5 Department of Medicine, SUNY Upstate Medical University, Syracuse, NY 13210, USA; 6 Department of Kinesiology and Nutrition, University of Illinois Chicago, Chicago, IL 60612, USA; 7 Department of Nutrition, Harvard T. H. Chan School of Public Health, Boston, MA 02115, USA; 8 Division of Nutritional Sciences, College of Human Ecology, Cornell University, Ithaca, NY 14850, USA; 9 Department of Medicine (Geriatrics and Cardiology), University of Pittsburgh, Pittsburgh, PA 15261, USA; 10 Geriatric Research Education and Clinical Center (GRECC), VA Pittsburgh Healthcare System, Pittsburgh, PA 15240, USA

**Keywords:** spinal cord injury (SCI), time-restricted eating (TRE), intermittent fasting, obesity, cardiometabolic health, continuous glucose monitoring, glucose metabolism

## Abstract

**Background**: Veterans with spinal cord injury (SCI) represent the largest single group of individuals with SCI nationwide. They have a high prevalence of obesity (76.7%) and cardiometabolic disease (57.5%). Time-restricted eating (TRE) involves eating within a consistent, daily window of ≤10 h and can improve cardiometabolic health. Here, we investigated the feasibility and acceptability of TRE among veterans with SCI and obesity and collected preliminary data on its cardiometabolic effects. **Methods**: We implemented a 6-week, single-arm trial of 10 h daily TRE among a convenience sample of 15 veterans with SCI and obesity. **Results**: Fourteen participants completed the trial. Among completers, TRE had high feasibility (83.0% adherence; mean window of 9.2 ± 1.5 h) and moderately high acceptability (50% planned to continue TRE post-trial and 79% would recommend TRE to a friend). TRE also improved several markers of glycemic control, including 24 h glucose levels (−7 ± 3 mg/dL, *p* = 0.02); Glucose Management Indicator (GMI), which estimates average HbA1c levels (−0.2 ± 0.1%, *p* = 0.02); and time in range (3.2 ± 1.6%, *p* = 0.02); and reduced time above range (level 1 hyperglycemia) (−2.0 ± 0.9%, *p* = 0.04). Half of participants reported improvements in aspects of physical or mental health, including mood, energy, and quality of life. **Conclusions**: TRE is both feasible and acceptable to veterans with SCI and obesity and has the potential to improve glycemic control. Larger trials are needed to test the effectiveness of TRE for reducing weight and improving cardiometabolic health in this high-risk population.

## 1. Introduction

Obesity develops rapidly in individuals after a spinal cord injury (SCI) and impacts between 77 and 97% of the total SCI population, based on data as recent as 2021 [1,2]. Significant weight gain occurs within the first year post-injury. Lean mass is lost and is followed by the accumulation of adipose tissue [3,4,5,6]. Obesity, in turn, dramatically increases the risk of cardiometabolic disease [7,8,9,10], now one of the leading causes of mortality following SCI [11].

The largest group of individuals with SCI nationwide is veterans [12]. Veterans with SCI have a very high prevalence of obesity: 76.7% [1] compared to 41% of veterans overall [13] and 40.3% of American adults [14]. Veterans with SCI also have a high prevalence of obesity-associated comorbidities, particularly cardiometabolic disease. Based on published data in 2019, about half (49.7%) have type 2 diabetes, 55.1% have hypertension, and 57.5% have metabolic syndrome [1]. Obesity also adversely affects quality of life, contributing directly to pain and depression after SCI [15]. Interventions that reduce the risk of obesity and cardiometabolic disease, as well as improve mood and quality of life, are greatly needed.

One new approach to losing weight is to change when people eat, particularly their eating windows, defined as the daily period when calories are consumed [16,17]. Several clinical trials report that time-restricted eating (TRE), or eating within a consistent daily window of ≤10 h, induces weight loss and may improve cardiometabolic health, mood, fatigue, and physical function [18,19,20,21,22,23,24,25,26,27,28]. TRE, the most popular form of intermittent fasting [19], asks people to watch the clock instead of counting calories [20]. Despite no calorie counting, TRE leads to an inadvertent 5–20% calorie restriction, translating into modest weight loss, typically 3–5% after a few months [18,19,20,21,22,23,25,26]. Several trials also suggest that it may reduce blood pressure, lipids, and inflammatory markers, and improve glucose tolerance [19,20,25,27,29].

TRE offers potential advantages relative to conventional caloric restriction, which is often considered the gold-standard dietary treatment [30]. These advantages include better adherence, long-term sustainability, and little rebound weight gain in long-term practitioners [31,32]. TRE may also improve mood, reduce fatigue, and decrease the severity of depressive symptoms [22,28]. TRE may be particularly attractive to individuals with SCI. SCI results in systemic effects that significantly alter the metabolic milieu. SCI disrupts energy balance and induces chronic, low-grade inflammation, which may contribute to the high prevalence and early onset of obesity post-injury [33]. SCI also increases the risk of insulin resistance [34] and leads to the loss of skeletal muscle—tissue that normally plays an important role in utilizing glucose and acting as a metabolic sink [5]. In addition, SCI alters bodily cues and promotes diminished feelings of fullness [35], which could result in increased eating activity and weight gain. Thus, individuals with SCI may respond uniquely to TRE as both a behavioral intervention that limits eating windows and eating activity and as a dietary intervention that reduces cardiometabolic risk through metabolic alteration. TRE also appears to improve cardiorespiratory fitness and mobility [36] even in the absence of exercise, which would be important in a population with impaired mobility and reduced exercise options [10].

In a sample of veterans with SCI and obesity, the median eating window was 10.8 h [37]. Yet there was substantial variation in individual windows, which ranged from 7.0 to 14.5 h. Thus, a large fraction of individuals with SCI and obesity could potentially benefit from a shorter eating window. Yet despite TRE’s popularity among the lay public and its utility in research as a weight-loss intervention, TRE has not been previously investigated in the SCI population. Therefore, we conducted a 6-week pilot and feasibility study of 10 h TRE in veterans with SCI and obesity. We hypothesized that 10 h TRE would be both feasible (adherence > 80%) and acceptable (based on survey feedback), without leading to any serious adverse events.

## 2. Materials and Methods

### 2.1. Overview

We conducted a 6-week, single-arm study to assess the feasibility and acceptability of TRE and to collect preliminary data on its cardiometabolic effects in veterans with SCI. We included no control group. The study was conducted according to the guidelines of the Declaration of Helsinki and approved by the Institutional Review Board at the Syracuse Veterans Affairs (VA) Medical Center (protocol number: 1753882; approval date: 21 August 2023) and registered at ClinicalTrials.gov (NCT05921487). Written informed consent was obtained from all participants involved in the study by the lead author prior to study participation. Veterans were compensated $300 for completing the study.

### 2.2. Recruitment

We recruited a convenience sample of veterans with SCI seeking standard clinical care at a single VA SCI Center. The SCI system of care consists of Hub-and-Spoke networks with 25 Centers (“hubs”) located throughout the nation that provide rehabilitation, primary care, and specialty care for veterans with SCI. SCI Centers are supported by 122 designated medical facilities (“spokes”) that provide ancillary care closer to veterans’ homes [7,12]. Candidates in this study were recruited by SCI clinical providers from both the main center hub and its surrounding spoke clinic sites. Recruitment occurred between December 2023 and July 2024. Recruitment was rolling, and participants completed 2-week baseline studies of eating windows [37] prior to active enrollment in TRE, accounting for a delay in the start of the intervention.

We recruited veterans aged 18–89 years with chronic SCI (≥1 year post-injury), paraplegia (injury levels T1–S5), American Spinal Injury Association (ASIA) Impairment Scale grades A–D, and obesity (BMI > 22.0 kg/m^2^, an SCI-specific BMI cutoff) [38]. Veterans with either traumatic or non-traumatic SCI etiologies were eligible. Participants also had to have a baseline eating window of at least 10 h.

Exclusion criteria included: pregnant, planning to become pregnant in the next six months, or breast-feeding; heart failure; diabetes and prescribed insulin, sulfonylureas, or meglitinides (medications with high incidence of hypoglycemia); end-stage renal disease; dementia; cancer; advanced liver failure; porphyria; uncontrolled hyperthyroidism; orthostatic hypotension and prescribed midodrine or fludrocortisone; syncopal episode or significant weight loss (>10% body weight) in the past month; active suicidal ideation within the past six months; and acute medical issues such as sepsis. Veterans currently prescribed oral corticosteroids or weight loss medications, including glucagon-like peptide-1 receptor agonists and co-agonists (referred to as GLP-1 RAs; e.g., semaglutide and tirzepatide), were also excluded.

### 2.3. Intervention

After enrollment, baseline measures were obtained over a 2-week period designed to ensure weight stability. Thereafter, participants started the 6-week TRE intervention. Participants self-selected a 10 h eating window and were instructed to maintain this same eating window throughout the study. We provided verbal and written instructions on which foods and beverages constituted starting and ending the eating window. Water, black coffee, and unsweetened tea were permitted during the fasting period, whereas food additives like sweeteners and electrolytes were not. There were no additional dietary instructions or restrictions, and veterans could eat their preferred diet. Veterans were encouraged to drink plenty of water during the fasting periods and maintain their normal physical activity levels. After 6 weeks, veterans returned to the clinic for a post-intervention evaluation. All participants completed the TRE intervention between October 2024 and March 2025.

### 2.4. Safety and Adverse Events

Veterans with SCI were instructed to report safety-related concerns to the research team, clinicians, nurses, and home care specialists within the VA SCI system of care. We used standard rules for clinical trials to define adverse and serious adverse events (e.g., defining serious adverse events as any that result in death, are life-threatening, result in initial or prolonged hospitalization, disability or permanent damage, cause an anomaly/birth defect, require intervention to prevent permanent impairment or damage, or cause other serious/important medical event). Additionally, participants were queried at the trial’s conclusion about safety and adverse events, using a Diet Satisfaction Survey for event description and classification. We assessed albumin and prealbumin levels using a metabolic panel as one indicator of nutrition status [39]. We measured grip strength as an indicator of physical function at baseline and post-intervention.

### 2.5. Demographics and Medical History

Demographic and injury characteristics were obtained using the SCI/Disorders Registry, a comprehensive database maintained by the VA [12]. We collected the following information: sex, age, body mass index (BMI), race, ethnicity, living location (rural vs. urban), employment status, home setting (living alone vs. living with spouse/other adult), caregiver status, education level, SCI involvement (paraplegia vs. tetraplegia), injury duration, SCI etiology (traumatic vs. nontraumatic), neurological level of injury, and ASIA impairment scale, which categorizes injuries based on motor and sensory involvement and completeness of injury.

### 2.6. Outcomes

The primary outcomes were adherence and acceptability. As secondary outcomes, we assessed changes in several cardiometabolic endpoints, including body weight, waist circumference, blood pressure, heart rate, lipids (total cholesterol, triglycerides, high-density lipoprotein [HDL] cholesterol, and low-density lipoprotein [LDL] cholesterol), inflammatory markers (C-reactive protein [CRP] and erythrocyte sedimentation rate [ESR]), and glycemic control, including 24 h glucose levels, GMI (Glucose Management Indicator), and time in range. Secondary outcomes were assessed at baseline and post-intervention following a 10 h overnight fast.

### 2.7. Adherence

Veterans self-reported what times they started and stopped eating and drinking (>0 kcals) on a paper log for 2 consecutive weeks at baseline and throughout the 6-week intervention. Adherence was quantified as the percent of days that participants ate within 10 h and 10.5 h windows (30 min grace period).

### 2.8. Acceptability

At the end of the intervention, participants completed a Diet Satisfaction Survey. The survey was adapted from Lee et al.’s study of determinants of adherence in TRE in older adults [40] and augmented with questions based on key facilitators and barriers to TRE [16]. The survey consisted of 25 items, which were scored on a 5-point Likert scale (1 = strongly disagree, 5 = strongly agree). Twenty-two items addressed barriers and facilitators of TRE within three domains (biological, psychological, and social), while an additional three items evaluated participants’ overall experience with TRE.

### 2.9. Anthropometrics

Height was measured at baseline only, whereas all other anthropometric endpoints were measured at baseline and post-intervention. Measurements were obtained by a trained SCI nurse who followed standard protocols. Height was measured with a non-stretchable tape measure, with ambulatory veterans standing upright and non-ambulatory veterans lying supine. Waist circumference was measured at the level of the iliac crest at the end of a normal expiration, with ambulatory veterans standing upright and non-ambulatory veterans lying supine. Body weight was measured in a standard gown after voiding, using a conventional digital scale (Hopkins^®^ EZ Carry 440 LB Digital Scale; Caledonia, MI, USA) for ambulatory veterans and a calibrated wheelchair scale (Healthweigh^®^ Rice Lake Digital Wheelchair Scale; Rice Lake, WI, USA) for non-ambulatory veterans. For the latter, the weight of the wheelchair was first obtained, followed by the weight of the veteran in the wheelchair; then the figures were subtracted to obtain the weight of the veteran. One participant with healing wounds was instead evaluated on the Hill-Rom^®^ CareAssistTM ES Bed at baseline and post-intervention, following the manufacturer’s instructions (Baxter International; Deerfield, IL, USA).

### 2.10. Blood Pressure and Heart Rate

Blood pressure and heart rate were measured manually in duplicate after a 5 min rest in a quiet room, with a one-minute break between measurements. Blood pressure measurements were obtained using an aneroid sphygmomanometer (Welch Allyn^®^ Model 767; Baxter International; Deerfield, IL, USA). Participants were positioned in an upright (seated) position with the back and arm supported, and the arm at heart level. Measured values were averaged.

### 2.11. Continuous Glucose Monitoring

Veterans were fitted with continuous glucose monitoring (CGM) devices (Dexcom G6Pro; Dexcom Inc.; San Diego, CA, USA) to track 24 h glucose profiles. CGMs were inserted in the abdomen for all participants following the manufacturer’s instructions and used for 10 days during the baseline period and once during the TRE intervention. Participants were instructed to wear the sensor for 10 days during weeks 3–6 of the intervention. We selected this time frame to give participants an initial transition period to TRE to facilitate adherence [41]. One participant misunderstood and wore the sensor earlier (data included). One participant’s sensor was lost in transit (baseline measures). His interventional data were not included. Participants were blinded to all CGM data. Outcomes included 24 h mean glucose levels, GMI (which estimates average HbA1c levels), time in range, time above range, and time below range.

### 2.12. Serum Chemistry

We measured lipids (total cholesterol, triglycerides, HDL cholesterol, and LDL cholesterol); the inflammatory markers CRP and ESR; fasting glucose; and albumin and prealbumin. LDL cholesterol was measured directly and not calculated. All blood samples were collected at the VA Syracuse Medical Center using standard laboratory methods and procedures and analyzed with a chemical analyzer (Siemens Atellica CH 930 Analyzer, Siemens Medical Solutions USA, Inc., Malvern, PA, USA), with the exception of ESR (ALCOR^®^ iSED ESR Analyzer, ALCOR^®^ Scientific, Smithfield, RI, USA). All samples were analyzed on-site at the VA Syracuse Medical Center, except prealbumin, analyzed at the Buffalo VA Medical Center Core Laboratory.

### 2.13. Physical Function

Handgrip strength can be used in the SCI population with paraplegia to assess maintenance of function [42] and is widely used to indicate functional status and predict future functional limitations in the non-SCI population [43]. At baseline and post-intervention, participants were asked to squeeze the dynamometer (JAMAR^®^ Hydraulic Hand Dynamometer, Model J00105, Lafayette Instrument Company; Lafayette, IN, USA) with their dominant hand with as much force as possible and hold their grip for 3–5 s. Verbal encouragement was given by the research team during the assessment [44]. Three measures were obtained, and the average value was used.

### 2.14. Sample Size

Our work was modeled on a feasibility study of TRE in people with multiple sclerosis by Wingo and colleagues [45]. We targeted enrolling 15 participants to achieve a minimum sample of 12 completers, assuming up to 20% attrition. Fourteen participants ultimately completed the intervention.

### 2.15. Statistical Analyses

All analyses were conducted by a statistician using R statistical software (Version 4.4.2; R Core Team, 2024) and a two-sided significance threshold of α = 0.05. As this is a pilot and feasibility study, we used a threshold of α = 0.20 to flag outcomes particularly worth retesting in future larger clinical trials, referring to them as having numerically increased or decreased. This threshold is consistent with recommendations for pilot studies in clinical research in which an alpha as high as 0.25 is suggested [46,47]. This approach balances the trade-offs between type I and type II errors, and appropriately minimizes the risk of rejecting treatment options “that offer large patient benefits” [46]. In selecting an alpha of 0.20, we note that these results are exploratory.

Outcomes were analyzed in completers, as no interventional data were made available to the research team from the one participant who withdrew. Descriptive statistics were used to report adherence and acceptability data. Adherence data were reported as mean ± SD and were analyzed overall, by study week, weekday vs. weekend, and by day of the week. Acceptability data from the Diet Satisfaction Survey are summarized using the median and interquartile range (IQR). Data were analyzed by question item, grouped into an overall assessment (3 survey items), and grouped by domain (biological, psychological, social). Within each domain, items were classified as barriers or facilitators. Combined summaries were created for overall items and separately for facilitators and barriers.

For secondary and other outcomes, participants with missing values were excluded. One participant’s data were excluded from the analysis of glycemic endpoints due to having recently stopped GLP-1 RA medication only 1 week before starting the TRE trial. Change scores (post-pre) were assessed for normality using the Shapiro–Wilk test. Normally distributed data were analyzed using paired t-tests. If data were not normally distributed (*p* < 0.05), we used Wilcoxon signed-rank tests. For each endpoint, we report change scores, ± SEM, and *p*-values. For paired pre-post comparisons, we report Cohen’s d, calculated as the mean within-participant change divided by the standard deviation of the within-participant change scores. Given the pilot nature of this study and limited sample size, all *p*-values are unadjusted for multiple comparisons.

## 3. Results

### 3.1. Participant Characteristics

**Figure 1:**
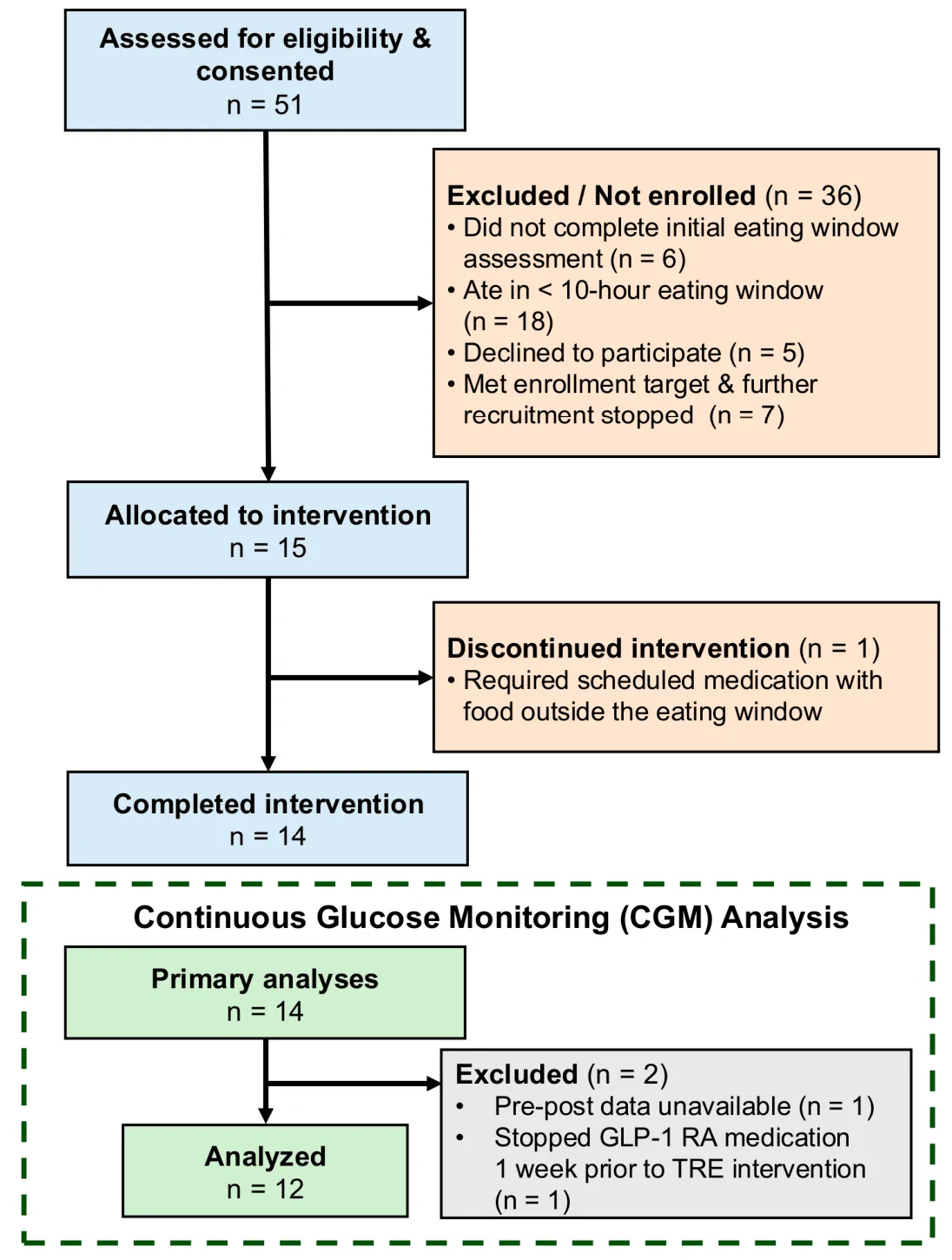
Participant flow diagram through the stages of enrollment, allocation, follow-up, and Analysis.

We consented 51 veterans, 45 of whom completed an initial eating window assessment. Of these, 27 had mean eating windows of at least 10 h. Twenty individuals were approached to enroll. Five declined, had moved, or were in the process of moving, and 15 enrolled, at which time recruitment was stopped (Figure 1). The sample was predominantly male, white, and non-Hispanic (>85% in all categories) (Table 1). Participants had a mean age of 62 ± 9 years and a mean BMI of 32.4 ± 5.7 kg/m^2^. The mean injury duration was 21.9 ± 16.3 years. Forty percent of participants had motor complete injuries, while 60% had motor incomplete injuries. The mean baseline eating period was 12.3 ± 2.1 h, with a mean start time of 08:51 ± 02:12 and a mean end time of 19:45 ± 05:09. Ultimately, one participant withdrew from the study, citing the need to take his anti-spasticity medication (which was scheduled during his fasting window) with food to maintain efficacy of treatment. Fourteen participants completed the trial, translating into a retention rate of 93%.

### 3.2. Adherence

Self-selected start times ranged from 08:00 to 12:00, with 09:00 as the most common start time (5/14, 36%), followed by 08:00 (3/14, 21%) (Table 2). Participants were scheduled to follow TRE across 588 person-days. Two missed person-days (0.34%) were excluded, resulting in 586 analyzable person-days. During the intervention, participants ate within a mean window of 9.2 ± 1.5 h, which corresponds to a 3.1 ± 1.5 h reduction in the length of the eating window (*p* < 0.001). Twelve out of 14 participants (86%) delayed the start time of eating. All participants stopped eating earlier in the evening, by 2.0 ± 0.3 h on average; the median shift was 1.8 h earlier (IQR, 1.3, 2.4; *p* = 0.001).

**Figure 2:**
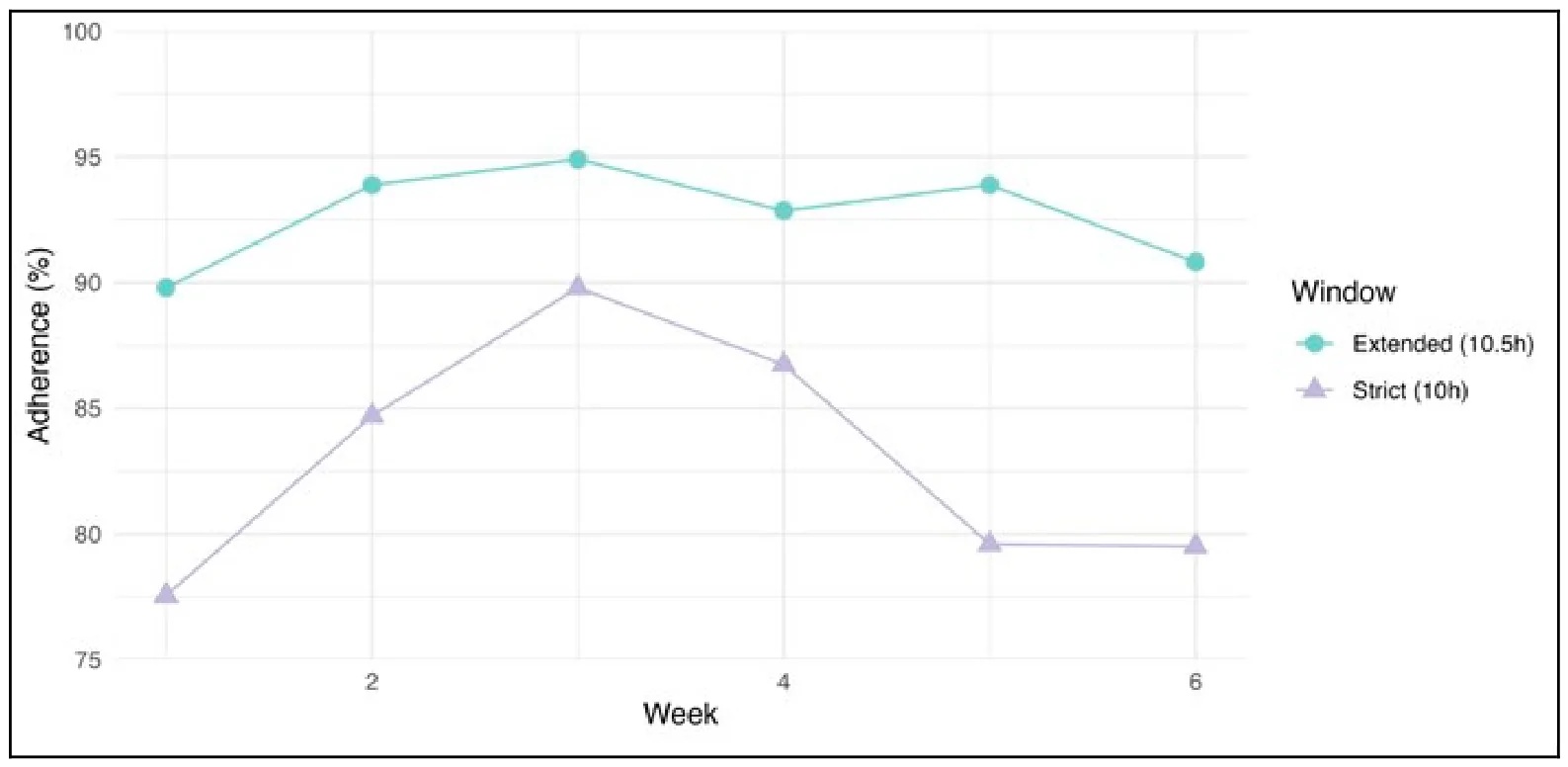
Weekly adherence to TRE windows.

Adherence to the 10 h window was 83.0% overall. With a 30 min grace period, adherence to a 10.5 h window was 92.7%. Weekly adherence to a 10 h window increased from 77.6% in week 1 to a peak of 89.8% in week 3 and then returned to approximately 80% in weeks 5–6 (Figure 2). In contrast, adherence to a 10.5 h window was a bit more stable across the 6-week study, ranging from 89.8% to 94.9%. Adherence patterns remained similar across weekdays and weekends, though adherence did vary across individual days of the week. Adherence to a 10 h window was highest on Mondays (89.3%) and Fridays (86.9%), and lowest mid-week (78.6% on both Wednesday and Thursday). There was comparatively smaller variation across days of the week for a 10.5 h window, with adherence ranging from 89.3% to 95.2%.

### 3.3. Acceptability

**Figure 3:**
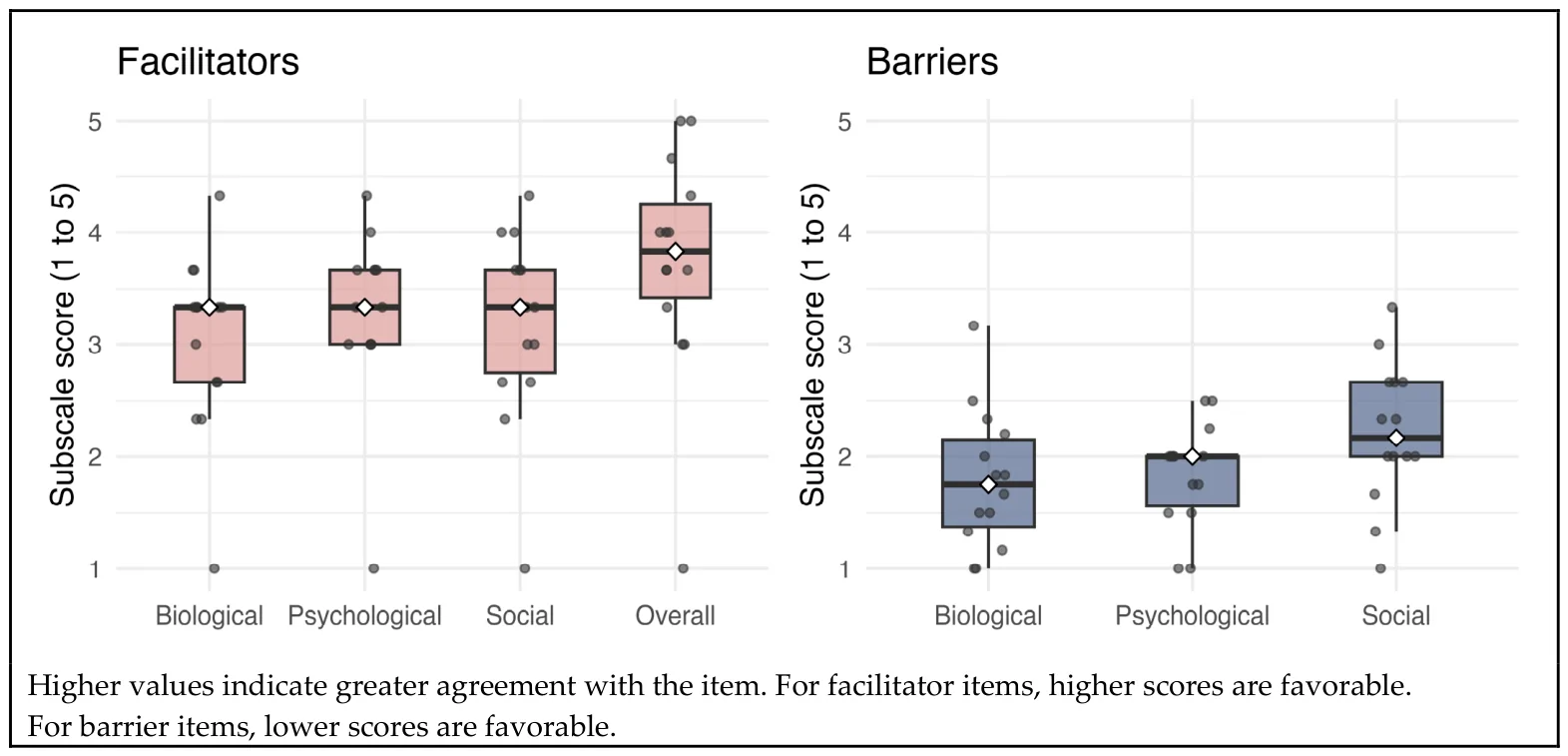
Diet Satisfaction Survey results by category and domain.

Participants completed a Diet Satisfaction Survey post-study to assess their overall experience with TRE and to report perceived barriers and facilitators within biological, psychological, and social domains (Table 3 and Figure 3). Seventy-nine percent (11/14) would participate in the study again, and 79% (11/14) would recommend TRE to a friend. When asked about plans to continue fasting, 7 out of 14 (50%) participants answered “agree” or “strongly agree.” An additional 4 out of 14 rated the item “neutral.” Fifty percent (7/14) reported benefit in at least one aspect of physical or mental health, responding positively (“agree” or “strongly agree”) to one or more of the following items: more energy than usual, improved overall physical health, improved mood, and improved overall quality of life.

Barriers aggregated across biological, psychological, and social domains had a relatively low median score of 2.00 (IQR 1.54 to 2.27), equivalent to a rating of “disagree.” Barriers were lowest for the biological domain (1.75, IQR 1.38 to 2.15) and highest for the social domain (2.17, IQR 2.00 to 2.67). On individual items, 86% (12/14) reported that TRE did not decrease energy levels, worsen mood, or negatively impact social life; 79% (11/14) reported that TRE did not make tasks at work or home more difficult or lead to significant hunger; and 71% (10/14) reported that TRE did not interfere with sleep.

Facilitators combined across biological, psychological, and social domains had a median of 3.28 (IQR 3.06 to 3.53), an assessment closer to “neutral.” Across individual domains, facilitators all had a median score of 3.33 (biological IQR 2.67 to 3.33; psychological IQR 3.00 to 3.67; and social IQR 2.75 to 3.67). Thirty-six percent (5/14) reported improvements in overall physical health, including body weight; 64% (9/14) reported that TRE fit in easily with their routines; 21% (3/14) experienced improved mood; and 7% had increased energy levels.

Participants adapted to TRE over time. Seventy-one percent (10/14) reported that TRE became easier as the study progressed, with an additional 21% (3/14) indicating no change over time. No participant (0%, 0/14) said that TRE became more difficult over time.

### 3.4. Adverse Events and Safety

There were no serious adverse events. Based on Diet Satisfaction Survey results, two participants reported more difficulty falling asleep, staying asleep, or waking up during fasting; two were uncomfortably hungry while fasting; and one noticed new digestion problems while fasting. Two participants reported worsening muscle spasms as a result of changing medication schedules; changes were made to anti-spasm medication to accommodate fasting periods. This resulted in one participant withdrawing. This participant also noted increased irritability and anxiety, which fully resolved after stopping TRE.

As safety endpoints reflecting nutritional status and physical function, albumin (0.06 ± 0.06 g/dL, *p* = 0.34), prealbumin (1.1 ± 1.2 mg/dL, *p* = 0.38), and grip strength (0.8 ± 2.3 kg, *p* = 0.73) did not change over the course of the study (Table 4).

### 3.5. Anthropometrics

Body weight did not change during the trial (0.1 ± 0.5 kg; *p* = 0.89), whereas waist circumference tended to numerically decrease by 1 ± 1 cm (*p* = 0.15; Table 4), an exploratory finding.

### 3.6. Cardiometabolic Health

As shown in Table 4, there were no significant changes in cardiovascular measures, including systolic blood pressure (*p* = 0.62), diastolic blood pressure (*p* = 0.21), heart rate (*p* = 0.91), triglycerides (*p* = 0.31), HDL cholesterol (*p* = 0.22), LDL cholesterol (*p* = 0.36), erythrocyte sedimentation rate (*p* = 0.79), and C-reactive protein (*p* = 0.27). However, total cholesterol numerically increased by 8 ± 5 mg/dL (*p* = 0.14), an additional exploratory finding.

Multiple markers of glycemic control improved and reached statistical significance at the α = 0.05 level. Twenty-four-hour glucose levels decreased by 7 ± 3 mg/dL (*p* = 0.02) and the GMI decreased by 0.2 ± 0.1% (*p* = 0.02), both with large effect sizes (d ~0.8). Time in range improved by 3.2 ± 1.6% (*p* = 0.02), and time above range (level 1) fell by 2.0 ± 0.9% (*p* = 0.04). Fasting blood glucose showed no significant change (*p* = 0.35).

## 4. Discussion

We conducted the first clinical trial of TRE in participants with SCI, as well as the first clinical trial of TRE in a veteran population. Results of our 6-week, single-arm study indicate that 10 h TRE is both feasible and acceptable among veterans with SCI and obesity, as well as safe. TRE also improved markers of glycemic control, including 24 h glucose levels and GMI, which estimates average HbA1c levels. We found high adherence to 10 h TRE, with participants adhering roughly 6 out of 7 days. The mean daily eating window was 9.2 h, and mean adherence was 83.0% to a 10 h window and 92.7% to a 10.5 h window. Safety was maintained throughout the trial. One participant withdrew from the study, translating into a retention rate of 93%.

TRE improved several markers of glycemic control, including GMI. The mean GMI at baseline was in the prediabetic range, so while the absolute change in GMI was modest (0.2%), the change constitutes a meaningful metabolic improvement, particularly in those with early dysglycemia. These findings seem to suggest that TRE has genuine glycemic benefits, though these results should be interpreted with caution. The fact that TRE may improve glycemia is notable in a population with a high prevalence of type 2 diabetes, nearly 50% [1]. In our sample, 36% had type 2 diabetes or prediabetes.

Mechanistically, TRE appears to improve glucose metabolism through multiple proposed pathways, enhancing insulin sensitivity and beta-cell function [19]. These glycemic improvements are seen even when TRE is matched against isocaloric diets, indicating that the benefits of TRE extend beyond energy restriction [19,48]. TRE also improves blood pressure and oxidative stress when matched against isocaloric diets, and is proposed to impact circadian rhythms and metabolic flexibility [19]. Thus, TRE may be well suited for individuals with SCI, who experience profound physiologic changes after SCI that increase the risk of cardiometabolic dysfunction [10,11], in particular dysglycemia and obesity [1,33].

TRE had moderately high acceptability among participants, as demonstrated by diet survey results. Half of participants had immediate plans to continue TRE post-study, and nearly 80% would recommend TRE to a friend. There were few reported barriers across biological, psychological, and social domains. Low perceived barriers may account for the participants’ enthusiasm for recommending TRE and for half planning to continue TRE. Barriers were lowest in the biological domain, indicating that challenges such as diet, digestion, energy, hunger, and sleep were either minor or not present. Social barriers ranked highest. Nonetheless, 86% of participants reported that TRE did not negatively impact their social lives. Instead, 36% of participants cited that it was “inconvenient” to restrict food intake to the designated window. Facilitators ranked closer to “neutral” (3 out of 5) across domains.

In addition to moderately high feasibility and acceptability, fifty percent of participants reported health benefits in at least one aspect of physical or mental health. Twenty-one percent reported improved quality of life and mood, and 36% reported that their overall physical health improved. Although participants reported health benefits, they did not lose weight. TRE consistently induces weight loss in comprehensive reviews and meta-analyses [19,20,27,32]. Perhaps 6 weeks was insufficient for a weight loss intervention among our participants. Jamshed and colleagues did not find statistically significant weight loss until week 8 in their TRE intervention [22], for example. Shorter eating windows may also promote weight loss more effectively. One meta-analysis found that ≤8 h windows were effective for losing weight, whereas longer windows were not, indicating that weight loss results with 10 h windows are more mixed [49].

Aside from glycemic control, we did not find significant benefits in other cardiometabolic risk factors, though the study was not powered to detect such changes. TRE numerically improved waist circumference (*p* = 0.15), an exploratory result which may indicate some loss of abdominal fat over the 6 weeks, though body composition was not directly measured. TRE also appeared to numerically raise total cholesterol by 8 ± 5 mg/dL (*p* = 0.14), an additional exploratory result. Lipids have worsened in some trials of late TRE, including one-meal-a-day protocols [50]. Although a comprehensive review of TRE trials reported no net effects on lipids [19], a recent subgroup analysis found that TRE increases LDL cholesterol in patients with severe obesity (BMI ≥ 40 kg/m^2^), whereas it appears to have a neutral or positive effect in patients with non-severe obesity (BMI 30–40 kg/m^2^) [51]. While cutoffs for severe and non-severe obesity within SCI populations have not been established, study participants had a mean BMI of 32.4 kg/m^2^, significantly greater than the BMI cutoff for obesity after SCI (>22 kg/m^2^), and possibly more akin to a severe degree of SCI obesity. Despite meta-analyses reporting that TRE reduces blood pressure, we found no changes in blood pressure.

Mitigating obesity and its cardiometabolic consequences remains a priority after SCI, and our work demonstrates strong interest among veterans with SCI in weight loss interventions, including TRE. Nearly 10% (45/464) of our local SCI population completed baseline assessments of eating windows in preparation for this trial, and we easily met prespecified target enrollment goals. Our work revealed unique factors that could impact adherence to TRE. Eating patterns, for example, show substantial variation after SCI, with eating windows ranging from 7.0 to 14.5 h at baseline [37]. This may be due, in part, to the broad yet heterogeneous impact of SCI on biology and behavior. Moreover, among our study participants, self-selected eating times showed significant variation, with windows starting between 08:00 and noon. Self-selected eating windows may be most appropriate to accommodate this diversity, yet may limit some of the cardiometabolic benefits of eating earlier in the day [19,52]. Nonetheless, all study participants stopped eating earlier in the evening, by 2.0 ± 0.3 h on average, which could prove beneficial for health. Indeed, many reported snacking less and drinking less alcohol after dinner.

Among those with SCI, polypharmacy may be a potential barrier to adherence to TRE. One participant withdrew from the study due to his medication regimen. Polypharmacy is common after SCI, and a majority of individuals with SCI meet various criteria for polypharmacy [53,54]. Medications such as pain and anti-spasticity regimens are often taken multiple times throughout the day and often with food [53]. This barrier can be managed in many cases but may require some trial-and-error on behalf of participants and providers to adjust drug schedules during TRE.

### 4.1. Future Perspectives

Our findings suggest several promising directions for future research. Longer, well-powered, randomized controlled studies that examine weight loss as well as glycemic endpoints are warranted among veterans with SCI. These studies are critical to determine the efficacy of TRE treatment, in particular in individuals with prediabetes and type 2 diabetes. As this was a pilot study, α = 0.2 was used as a threshold to retest endpoints in a larger trial [46,47], suggesting that it is worth retesting the effects of TRE on waist circumference and lipids as well. In line with recommendations to optimize TRE human trials [48], trial duration should be extended to at least 3 months and preferably greater than 6 months. With this longer time frame, researchers can more accurately assess weight changes and changes in clinically significant endpoints (like HbA1c as a measure of glycemic control), as well as sustainability of TRE treatment for cardiometabolic risk reduction. Integrating CGM into this work may allow researchers to clarify how TRE influences glucose metabolism after SCI. Future work can also include assessment of dietary intake, as energy restriction is a key driver of weight loss in TRE studies, as well as assessment of physical activity [48], in order to better distinguish the independent effects of TRE from those of caloric intake and other lifestyle factors.

Additionally, since weight loss may result in an unfavorable lean mass loss, the quality of weight loss should be assessed. In the SCI population, additional lean mass loss could have disproportionately adverse consequences on function, frailty, and quality of life, and future work can test the effects of TRE on body composition (e.g., fat and lean mass). While TRE is associated with some loss of lean mass (~17% according to one recent systematic review and meta-analysis), this loss is considered “relatively small” and safely tolerated in general populations [49]. This loss is also similar to other dietary interventions, in which three-quarters of weight loss is adipose tissue and one-quarter is lean mass [55]. Yet it remains unknown whether these findings translate to the SCI population [55]. Moreover, more diverse SCI populations can be enrolled, with a greater proportion of participants who are young, female, and from racially and ethnically diverse backgrounds, which may allow researchers to identify subgroups that derive significant benefit from TRE, informing future clinical practice guidelines and personalized dietary therapy for veterans and other persons with SCI.

### 4.2. Limitations

Despite the novelty and strengths of our study, this work has several limitations. Foremost, this is a single-arm trial, which limits the ability to infer causal benefits of TRE. This does not limit our findings about feasibility and acceptability. Second, we excluded those with very significant cardiometabolic disease to ensure safety while fasting. This limits generalizability, given the high prevalence of cardiometabolic disease among veterans with SCI [1]. Third, the sample size was small, and the duration of the intervention was short, which limited our ability to detect significant changes in secondary outcomes, including weight loss and cardiometabolic outcomes. Fourth, our sample was predominantly male, white, non-Hispanic, and over the age of 60 years. This demographic captures our intended study population, veterans with SCI, and is consistent with both local and national demographic data [56]. However, it does not reflect the broader (non-veteran) SCI population, which includes a greater percent of individuals who are younger, female (>20%), Black (>20%), and Hispanic (>10%) [57,58,59]. Fifth, we limited enrollment to those with paraplegia. This was intentional to reduce neurological heterogeneity. Nonetheless, obesity prevalence is greater in those with tetraplegia [11,60], and further investigation of TRE in this group is warranted. Sixth, we did not pre-specify a washout period between discontinuation of GLP-1-based therapy and initiation of TRE. Two study participants discontinued GLP-1 RAs to participate in the study (the rest were GLP-1 RA-naïve), and one did so within 4–5 half-lives of last medication use. “[A]fter 4 to 5 half-lives, the plasma concentration of a given drug typically falls below a clinically relevant concentration and is considered effectively eliminated” [61]. Nonetheless, this resulted in the exclusion of one participant’s CGM data, adding caution in interpretation of glycemic results. Seventh, we did not measure dietary intake, and cannot determine if glycemic improvement is primarily linked to TRE or caloric restriction. Eighth, we did not measure physical activity levels and cannot assess the extent to which this factor may have impacted results. Ninth, *p*-values were not adjusted for multiple comparisons. This approach is consistent with the exploratory nature of pilot studies, though it increases the risk of false-positive findings. Tenth, our food log data were self-reported. While self-reported instruments may be inaccurate, we believe data inaccuracies have been minimized, as only start and stop times of eating were reported. This task is straightforward and less prone to inaccuracies than self-reported energy intake [62]. Eleventh, study participants receive SCI care through the VA. To facilitate social desirability bias, participants may have tended to be more positive about their TRE experiences in post-study surveys. Finally, we did not assess body composition and cannot determine how TRE affects retention of lean mass in this unique population with limited physical mobility. Importantly, TRE did not negatively affect nutritional status or muscular strength, as measured by grip strength.

## 5. Conclusions

In a sample of veterans with SCI and obesity, we found that TRE is feasible, acceptable, and safe, and improves glycemic control. We found evidence suggesting that it is worth retesting whether TRE may reduce waist circumference, even in the absence of weight loss, in a larger clinical trial. Fifty percent of participants reported improvements in aspects of physical or mental health, including mood, energy, and quality of life, and half had immediate plans to continue TRE post-study. This indicates that a large fraction of SCI participants found TRE to be worthwhile. Future studies should investigate TRE in a larger population of veterans with SCI to confirm its feasibility and test its efficacy to promote weight loss and improve cardiometabolic health.

## Figures and Tables

**Table 1:** Demographic and injury characteristics of participants.

Demographic Characteristic	N = 15 ^1^
Sex, Male	15 (100%)
Age (years)	62 ± 9
Body Mass Index (kg/m^2^)	32.4 ± 5.7
Race, White	15 (100%)
Ethnicity	
Hispanic or Latino	2 (13%)
Non-Hispanic or Non-Latino	13 (87%)
Location	
Rural	5 (33%)
Urban	10 (67%)
Home Setting	
Lives Alone	5 (33%)
Lives with Spouse or Other Adult	10 (67%)
Caregiver	
Caregiver	4 (27%)
No Caregiver	11 (73%)
Education Level	
Some College or Higher Education or Technical School	13 (87%)
No College	2 (13%)
Occupation Status	
Not Employed	9 (60%)
Employed	6 (40%)
**Injury Characteristic**	
Spinal Cord Injury Involvement	
Paraplegia	15 (100%)
Injury Duration (years)	21.9 ± 16.3
Neurological Level of Injury	
Lumbosacral Level	6 (40%)
Thoracic Level	9 (60%)
ASIA Impairment Scale	
Motor Complete (AIS A/B)	6 (40%)
Motor Incomplete (AIS C/D)	9 (60%)
Etiology of Spinal Cord Injury	
Traumatic SCI	8 (53%)
Non-Traumatic SCI	7 (47%)
**Eating Window Characteristics**	
Start Time	08:51 ± 2:12
Stop Time	19:45 ± 5:09
Average Eating Period (hours)	12.3 ± 2.1

^1^ n (%); Mean ± SD.

**Table 2:** Eating windows of participants.

Participant	Self-Selected 10 h Eating Window	Mean Eating Windows			
		Pre (Hours)	Post (Hours)	Post-Pre (Hours)	Post-Pre (%)
P01	12:00–22:00	12.5	7.6	−4.9	−39.3
P02	9:00–19:00	13.0	9.4	−3.7	−28.2
P03	10:00–20:00	12.7	9.5	−3.2	−24.9
P04	8:00–18:00	13.0	9.1	−3.9	−30.3
P05	8:00–18:00	11.1	9.1	−2.0	−18.0
P06	10:00–20:00	10.9	9.2	−1.7	−15.6
P07	9:00–19:00	11.5	9.3	−2.2	−18.7
P08	11:00–21:00	13.1	8.3	−4.8	−36.6
P09	10:30–20:30	10.4	9.6	−0.9	−8.2
P10	9:00–19:00	11.3	10.5	−0.8	−7.1
P11	9:00–19:00	12.3	9.6	−2.7	−21.9
P12	9:00–19:00	13.4	9.5	−3.9	−29.0
P13	9:30–19:30	14.8	9.7	−5.1	−34.7
P14	8:00–18:00	12.1	8.1	−4.0	−33.3

**Table 3:** Diet Satisfaction Survey results by question.

Questions	Strongly Disagree N (%)	Disagree N (%)	Neutral N (%)	Agree N (%)	Strongly Agree N (%)
**Biological**					
I had difficulty falling asleep, staying asleep, or waking during fasting.	8 (57)	2 (14)	2 (14)	2 (14)	0 (0)
I had less energy than usual or felt sluggish while fasting.	3 (21)	9 (64)	2 (14)	0 (0)	0 (0)
I had more energy than usual while fasting.	2 (14)	1 (7)	10 (71)	1 (7)	0 (0)
I lost weight which made me want to continue fasting.	2 (14)	1 (7)	6 (43)	4 (29)	1 (7)
I noticed new digestion problems while fasting.	7 (54)	2 (15)	3 (23)	1 (8)	0 (0)
I was uncomfortably hungry while fasting.	6 (43)	5 (36)	1 (7)	2 (14)	0 (0)
It was difficult to eat enough calories within the eating period.	7 (50)	6 (43)	0 (0)	1 (7)	0 (0)
My diet was inadequate during the eating window.	8 (57)	5 (36)	1 (7)	0 (0)	0 (0)
My overall physical health improved while fasting.	1 (7)	0 (0)	8 (57)	4 (29)	1 (7)
**Psychological**					
Fasting became easier as the study went on.	1 (7)	0 (0)	3 (21)	6 (43)	4 (29)
Fasting became more difficult as the study went on.	9 (64)	5 (36)	0 (0)	0 (0)	0 (0)
Fasting was difficult because I had to skip my regular meals and snacks.	3 (21)	7 (50)	3 (21)	1 (7)	0 (0)
Fasting was more difficult than other diets previously tried.	3 (21)	9 (64)	1 (7)	0 (0)	1 (7)
My mood improved while fasting.	2 (14)	0 (0)	9 (64)	3 (21)	0 (0)
My mood worsened while fasting.	5 (36)	7 (50)	2 (14)	0 (0)	0 (0)
My overall quality of life improved during the fasting diet.	1 (7)	0 (0)	10 (71)	3 (21)	0 (0)
**Social**					
Fasting periods made tasks at work and home more difficult.	5 (36)	6 (43)	2 (14)	0 (0)	1 (7)
Fasting periods negatively impacted my social life.	5 (36)	7 (50)	1 (7)	1 (7)	0 (0)
It was inconvenient to restrict my food intake to the designated time frame.	2 (14)	6 (43)	1 (7)	3 (21)	2 (14)
My schedule made it easy to maintain my fasting period.	2 (14)	2 (14)	4 (29)	5 (36)	1 (7)
Social support helped me maintain my fasting period.	1 (7)	0 (0)	10 (71)	2 (14)	1 (7)
The diet fit well with other aspects of my daily routine, like exercise and sleep.	2 (14)	1 (7)	2 (14)	8 (57)	1 (7)
**Overall**					
I plan to continue fasting after this study.	2 (14)	1 (7)	4 (29)	4 (29)	3 (21)
I would participate in this study again.	1 (7)	1 (7)	1 (7)	4 (29)	7 (50)
I would recommend fasting to a friend.	1 (7)	0 (0)	2 (14)	9 (64)	2 (14)

n = 14 for all items except “I noticed new digestion problems while fasting” (n = 13).

**Table 4:** Cardiometabolic and safety outcomes.

	N	Pre Mean ± SD	Post Mean ± SD	Change Mean ± SEM	Cohen’s d	*p* Value	SCI-Specific Target Values *
**Safety Outcomes**							
Albumin (g/dL)	14	3.68 ± 0.45	3.74 ± 0.32	0.06 ± 0.06	0.27	0.34	≥3.5
Prealbumin (mg/dL)	13	24.3 ± 5.2	25.4 ± 5.8	1.1 ± 1.2	0.25	0.38	≥11
Grip Strength (kg)	14	93.8 ± 25.4	94.6 ± 27.0	0.8 ± 2.3	0.10	0.73	
**Cardiometabolic Outcomes**							
Weight (kg)	14	101.7 ± 20.5	101.8 ± 20.8	0.1 ± 0.5	0.04	0.89	
Waist Circumference (cm)	14	110 ± 16	109 ± 15	−1 ± 1	0.41	0.15	
BMI (kg/m^2^)	14	34.1 ± 8.8	34.2 ± 8.9	0.0 ± 0.2	0.08	0.62	≤22
Systolic Blood Pressure (mm Hg)	14	120 ± 10	119 ± 14	−1 ± 3	0.14	0.62	<130
Diastolic Blood Pressure (mm Hg)	14	66 ± 7	69 ± 8	3 ± 2	0.35	0.21	<85
Heart Rate (bpm)	14	67 ± 10	67 ± 7	0 ± 1	0.03	0.91	
Total Cholesterol (mg/dL)	14	162 ± 43	170 ± 41	8 ± 5	0.42	0.14	
Triglycerides (mg/dL)	14	117 ± 59	128 ± 70	11 ± 10	0.28	0.31	≤150
HDL Cholesterol (mg/dL)	14	44 ± 11	46 ± 10	2 ± 1	0.35	0.22	≥40 (male)
LDL Cholesterol (mg/dL)	14	95 ± 42	99 ± 43	4 ± 4	0.25	0.36	
Erythrocyte Sedimentation Rate (mm/hour)	14	12 ± 10	12 ± 11	0 ± 3	0.04	0.79	
C-Reactive Protein (mg/L)	13	1.47 ± 2.58	0.75 ± 0.52	−0.72 ± 0.62	0.32	0.27	
**Continuous Glucose Monitoring**							
Mean 24 h Glucose Levels (mg/dL)	12	122 ± 20	114 ± 14	−7 ± 3	0.78	0.02	
Glucose Management Indicator (%)	12	6.2 ± 0.5	6.0 ± 0.3	−0.2 ± 0.1	0.76	0.02	
Fasting Blood Glucose (mg/dL)	12	99 ± 22	102 ± 27	3 ± 3	0.28	0.35	≤100
Standard deviation (mg/dL)	12	25 ± 12	22 ± 8	−2 ± 2	0.40	0.10	
Coefficient of variation (%)	12	19.6 ± 6.4	19.1 ± 5.2	−0.5 ± 1.1	0.14	0.63	
Time above range, level 1 (%)	12	4.5 ± 7.2	2.5 ± 4.4	−2.0 ± 0.9	0.63	0.04	
Time in range (%)	12	93.3 ± 10.2	96.6 ± 5.1	3.2 ± 1.6	0.60	0.02	
Time below range, level 1 (%)	12	1.0 ± 2.3	0.3 ± 0.9	−0.7 ± 0.4	0.46	0.09	

* Values are based on recommendations in [10] for reducing cardiometabolic disease risk after SCI and for minimum levels of albumin and prealbumin [39] to avoid malnutrition. Other SCI-specific target values have not been established.

## Data Availability

Because the study was completed among Veterans, we are acting in accordance with VA policy to protect Veteran information and will make the de-identified data available upon reasonable request.

## Abbreviations

The following abbreviations are used in this manuscript:

ASIA	American Spinal Injury Association
BMI	Body Mass Index
CGM	Continuous Glucose Monitoring
GLP-1 RA	Glucagon-Like Peptide-1 Receptor Agonist
GMI	Glucose Management Indicator
IQR	Interquartile Range
SCI	Spinal Cord Injury
SD	Standard Deviation
TRE	Time-Restricted Eating
VA	Veterans Affairs
